# The Electrical Activity of the Orbicularis Oris Muscle in Children with Down Syndrome—A Preliminary Study

**DOI:** 10.3390/jcm10235611

**Published:** 2021-11-29

**Authors:** Liliana Szyszka-Sommerfeld, Magdalena Sycińska-Dziarnowska, Krzysztof Woźniak, Monika Machoy, Sławomir Wilczyński, Anna Turkina, Gianrico Spagnuolo

**Affiliations:** 1 Department of Orthodontics, Pomeranian Medical University in Szczecin, Al. Powst. Wlkp. 72, 70111 Szczecin, Poland; liliana.szyszka@gmail.com (L.S.-S.); magdadziarnowska@gmail.com (M.S.-D.); krzysztof.wozniak@pum.edu.pl (K.W.); monika.machoy@pum.edu.pl (M.M.); 2 Department of Basic Biomedical Science, Medical University of Silesia, Katowice, 3 Kasztanowa Street, 41200 Sosnowiec, Poland; swilczynski@sum.edu.pl; 3 Institute of Dentistry, I. M. Sechenov First Moscow State Medical University, 119435 Moscow, Russia; anna@turkin.su; 4 Department of Neurosciences, Reproductive and Odontostomatological Sciences, University of Naples “Federico II”, 80131 Napoli, Italy

**Keywords:** disability, Down syndrome, muscle activity, oral health, special need patients, surface electromyography

## Abstract

The aim of this study was to assess the electrical activity of the superior (SOO) and inferior (IOO) orbicularis oris muscles in children with Down syndrome (DS) and in children without DS. After applying the inclusion and exclusion criteria, 30 subjects were eligible to participate in the later stages of the research—15 subjects with DS (mean age 10.1 ± 1.1) and 15 healthy controls (mean age 9.8 ± 1.0). The electrical potentials of the SOO and IOO muscles were recorded using a DAB-Bluetooth electromyography machine (Zebris Medical GmbH, Germany) during the following tasks: At clinical rest, saliva swallowing, lip protrusion, lip compression, and production of the syllable/pa/. The Mann–Whitney U test was conducted to compare the study results between the groups. An analysis of the electromyographical (EMG) recordings showed that the electrical activity of the orbicularis oris muscle in children with DS and lip incompetence was significantly higher compared to healthy children during saliva swallowing, lip compression, and when producing the syllable/pa/, and this may suggest greater muscular effort due to the need to seal the lips during these functional conditions.

## 1. Introduction

Down syndrome (DS) is a chromosomal anomaly caused by the presence of an extra chromosome in pair 21, which was originally described by John Langdon Haydon Down in 1866 [1]. This genetic disorder is associated with mental impairment at many levels, as well as with disruptions in dentofacial aesthetics and dysfunctions of the masticatory organ that may negatively impact the health and quality of life of DS patients [2,3,4]. As a consequence, individuals with Down syndrome constitute a group of patients with special oral health needs.

One of the most common characteristics of subjects with DS is the presence of generalized muscular hypotonia, which directly affects the stomatognathic system [1,2]. Generalized orofacial muscles hypotonia e.g., orbicularis oris, zygomatic, masseter, and temporalis muscles, contributes to poor oral seal, poor suck, weak tongue control, and problems with jaw stability. This may result in impairments to speech, swallowing, and mastication in these individuals [5,6]. Hypotonia, muscle weakness, sluggish motor responses, and sensory changes can reduce their ability to maintain proper muscle balance [5]. Other general manifestations found in subjects with DS are stunted growth, congenital heart defects, thyroid dysfunction, brachycephaly, upper airway obstruction, respiratory breathing disorders, severely inhibited sagittal development in the midface region, reduced size of maxillary and mandibular bones, breathing through the mouth, risk of aspiration pneumonia, and a weak immune system [2].

Common oral features in patients with DS include the delayed eruption of deciduous and permanent dentition, an open bite or crossbite, Class III malocclusion, hypodontia, microdontia, macroglossia, and reduced oral cavity providing oral breathing and salivary leak. DS individuals usually present with poor oral hygiene and periodontitis [5,7,8].

Considering the fact that orofacial muscle hypotonia is one of the main characteristics of patients with DS that can directly affect their systemic and oral health as well as their ability to interact socially, research on muscle activity in these subjects is essential. Hypotonia of the orbicularis oris muscle is usually detected during a clinical examination. However, the visual assessment of muscular hypotonia is often subjective and devoid of much relevant qualitative and quantitative information [9]. Nowadays, advances in different imaging techniques have expanded the diagnostic possibilities within the masticatory organ [10,11]. One of the few useful instrumental diagnostic methods available for assessing muscle activity is surface electromyography (sEMG) [12]. It makes it possible to evaluate muscle functioning in an objective and quantitative way by identifying their electrical potentials. The advantages of sEMG are its non-invasiveness, simplicity, and availability [13].

To date, only a few studies have been conducted on the electromyographical (EMG) activity of the orbicularis oris muscle in patients with DS [9]. In light of the above, research is needed on lip EMG muscle function in DS subjects. The identification of the electromyographic pattern of the orbicularis oris muscle may provide a basis for the development and implementation of more effective methods of therapy, and as a result, may contribute to achieving functional improvement in the masticatory organ in this group of patients, particularly in the case of DS children.

The aim of this study was to assess the electrical activity of the superior and inferior orbicularis oris muscles both in children with DS and in those without DS. Our hypothesis is that no differences exist between DS and healthy subjects with regard to the EMG potentials of the upper and lower lip at rest, during saliva swallowing, lip protrusion, lip compression, and when producing the syllable/pa/.

## 2. Materials and Methods

### 2.1. Ethical Approval

The study was conducted in accordance with the guidelines of the Helsinki Declaration and the protocol was approved by the Local Bioethics Committee of the Pomeranian Medical University in Szczecin, Poland (protocol number: KB-0012/08/15). All the children’s parents gave their written consent for the procedures to be performed.

### 2.2. Study Population

Seventy individuals between the ages of 7.9 and 11.8 were invited to participate in the study. After applying the inclusion and exclusion criteria, 30 subjects were eligible to participate in the later stages of the research—15 subjects with DS (mean age 10.1 ± 1.1) and 15 healthy controls (mean age 9.8 ± 1.0). All the subjects had mixed dentition and had to meet all the inclusion criteria listed below. Forty individuals who did not meet all listed inclusion criteria were excluded from the study groups.

The children with DS were selected from subjects who had been referred to the Developmental Facial Abnormalities Clinic in Szczecin, Poland between August and October 2019. All individuals with DS had lip incompetence. The following inclusion criteria for the DS group were applied: The presence of a Down syndrome diagnosis, female and male gender, mixed dentition, lip incompetence, Class I occlusion according to Angle’s classifications, no coexistence of systemic diseases affecting the muscles, no drugs taken that could affect muscle activity, no history of trauma or surgical treatment in the orofacial region, mental competence to participate in the research procedures, and voluntary consent to participate in the research. Subjects with primary or permanent dentition, as well as subjects with Angle Class II or Class III malocclusions, systemic diseases, taking medications that could affect muscle activity, a history of trauma or surgery in the orofacial area, and/or individuals who were mentally unable to participate in the study procedures were excluded from the DS group.

The control subjects were recruited from children who had been referred to the Orthodontics Outpatient Clinic in Szczecin, Poland between August and October 2019. The following inclusion criteria for the control group were applied: No presence of Down syndrome or other congenital diagnosis, female and male gender, mixed dentition, Class I occlusion with a proper relationship between the upper and the lower dental arches, normal lip seal, nose breathing, no diagnosis of atypical swallowing, no previous orthodontic treatment, no history of systemic diseases affecting the muscles, no drugs taken that could affect muscle activity, no history of trauma or surgical treatment in the orofacial region, and voluntary consent to participate in the research. Subjects with primary or permanent dentition, malocclusions, including Angle Class II or Class III malocclusions, as well as subjects with abnormal lip seal, mouth breathing, diagnosis of atypical swallowing, previous orthodontic treatment, systemic diseases, taking medications that could affect muscle function, and/or individuals with a history of orofacial trauma or surgery were excluded from the control group.

### 2.3. Experimental Procedures

All subjects underwent a thorough examination by a single experienced examiner. It consisted of two parts: A clinical examination and electromyographic assessment of the orbicularis oris muscle.

#### 2.3.1. Clinical Examination

First, the general medical history of each participant was analyzed. During the clinical examination, each subject was assessed as having competent lips (CL) when the lips were in light contact with no contraction of the mentalis muscle, or as having incompetent lips (IL) when the lips were apart at clinical rest or when the children’s lips were in contact but presenting increased activity of the mentalis muscle. The following were also evaluated: Mode of breathing, assessed via a questionnaire answered by the children’s parents, mouth posture, which was observed by an examiner, the condition of the ear, nose, and throat, as well as speech and language. Based on this assessment, the subjects were divided into two groups: Those predominantly breathing through the nose and those predominantly breathing through the mouth. Visual observation of the subjects’ perioral muscles was also used to diagnose atypical swallowing. Atypical swallowing was defined as anterior or lateral pressure of the tongue against the dental arches, or when the tongue is placed in an anterior or lateral position during swallowing. Occlusal features, including vertical overlap, overjet, Angle Class, crossbite, and open bite were examined via intraoral analysis of the dental arches in three planes. The subjects’ facial profiles were evaluated on the basis of specific anthropometric landmarks.

#### 2.3.2. Electromyographical Assessment of the Orbicularis Oris Muscle Activity

An EMG analysis of orbicularis oris muscle activity was performed using a DAB-Bluetooth electromyography machine (Zebris Medical GmbH, Isny im Allgäu, Germany) with a gain level of 1000 times, a high-pass filter of 7 Hz–5 kHz, a channel sampling rate of 1 kHz, and an analogue digital converter (12 bits of dynamic resolution range, input impedance for analogue channels of 146 kΩ).

**Figure 1:**
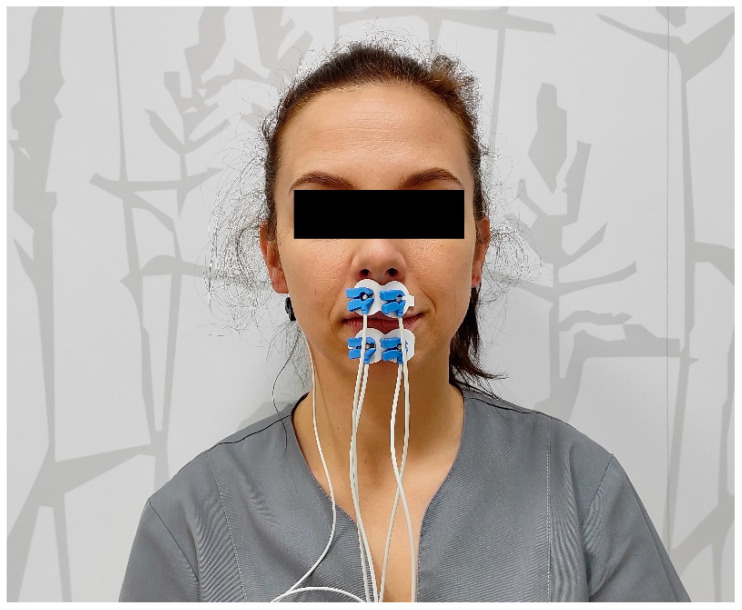
Location of electrodes (Noraxon Dual Electrode, Noraxon, Scottsdale, AZ, USA) during sEMG recordings of the superior orbicularis oris (SOO) and inferior orbicularis oris (IOO) muscles.

Bipolar surface electrodes (Ag/AgCl with a constant distance of 20 mm between the electrodes—Noraxon Dual Electrode, Noraxon, Scottsdale, AZ, USA) were attached to the superior and inferior orbicularis oris muscles. During the EMG recordings the subjects’ sat in a dentist’s chair with the head in a natural head position [14]. The electrodes were arranged as follows: An the superior orbicularis oris (SOO) muscle—along the line from the lip commissure to the nose (the subnasal point); the inferior orbicularis oris (IOO) muscle—along the line from the lip commissure to the mandibular midline; and the reference electrode—lower and behind the right ear [15,16] (Figure 1).

Each participant was prepared for the EMG recordings in the following manner: (1) The skin was cleansed with a 70% ethyl alcohol solution to reduce impedance; (2) after placement of the electrodes, an impedance test was performed using a Metex P-10 measuring device (Metex Instruments Corporation, Seoul, Korea) to confirm the area had been correctly prepared (skin impedance of 1 × 10^3^–30 × 10^3^ Ω). The sEMG examinations began 5 min later.

The electrical activity of the SOO and IOO muscles was recorded during the following sequence of experimental tasks: (1) At clinical rest when the lips were relaxed; (2) when swallowing saliva; (3) when the lips were in a protruding position; (4) when the lips were compressed; and (5) when producing the syllable/pa/. Before the sEMG measurements were taken, all the subjects were instructed in the procedure.

To ascertain stability, all the tasks were repeated at least three times. For each subject, all the EMG data were based on the arithmetic mean of the last two EMG recordings since the first EMG measurement frequently varied significantly from the other two repetitions and was not taken into consideration [17,18]. Between each action, the participants were instructed to relax for about 1 min to avoid muscle fatigue.

The signals from the electrodes were amplified, digitized, and stored on a personal computer. Then, the sEMG data were normalized in relation to the amplitude using the peak electromyographical value. Since the electromyographic signals exhibit considerable variability, their absolute values provide little information for comparisons to be made between individuals. Hence, the normalization procedure is important for the preliminary processing of the raw data so as to guarantee the reliability of further analyses. The situation with the highest potentials for all the tasks served as the maximum reference point for the muscle and had been taken as 100% of the muscle activity of the orbicularis oris muscle. EMG muscle activity during lip protrusion differed significantly from all other clinical conditions and showed the highest electromyographic activity of all. Finally, the normalization procedure involved expressing the measured data as a percentage of the reference value, i.e., lip protrusion, based on the following formula: Mean EMG values (µV) during condition/mean EMG values (µV) during lip protrusion × 100% (µV/µV%) [15,16].

The repeatability of the EMG protocol was tested by performing duplicate EMG recordings on 10 participants. The two independent measurements of muscle electrical activity were separated by 15 minutes’ rest between activities.

### 2.4. Statistical Analysis

The data were analyzed using STATISTICA 13.0 PL for Windows software package (StatSoft Poland, Cracow, Poland). Since the Shapiro–Wilk test revealed that the data were not distributed normally, non-parametric tests were carried out to provide a statistical analysis of the EMG scores. The Mann–Whitney U test was conducted to compare the study results between groups. A value of *p* < 0.05 was considered significant.

## 3. Results

Table 1 summarizes the characteristics of the study population. All the subjects with DS had an abnormal lip seal. Sixty-six percent of the DS subjects had atypical swallowing and 53.3% predominantly breathed through their mouth. Posterior crossbites were diagnosed in 66.7% of the DS subjects, while 33.3% of the subjects with DS were diagnosed with anterior crossbites, as well as with anterior open bites. Lateral open bites were diagnosed in 26.7% of the individuals with DS.

No significant differences (*p* > 0.05) were observed between the repeated sEMG recordings with regard to any of the analyzed variables (at rest, when swallowing saliva, compressing lips, and when producing the syllable/pa/).

**Figure 2:**
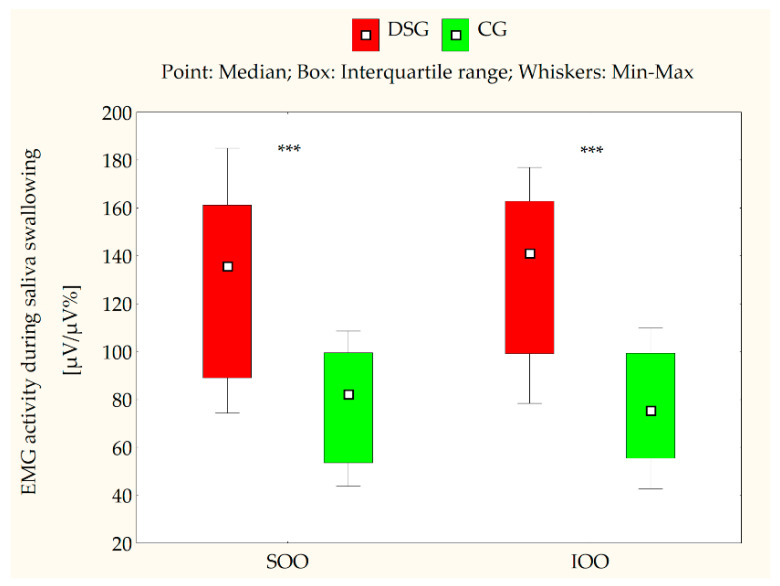
EMG activity of the superior orbicularis oris (SOO) and inferior orbicularis oris (IOO) muscles (µV/µV%) during saliva swallowing in the Down syndrome group (DSG) and the control group (CG); *** *p* < 0.001.

**Figure 3:**
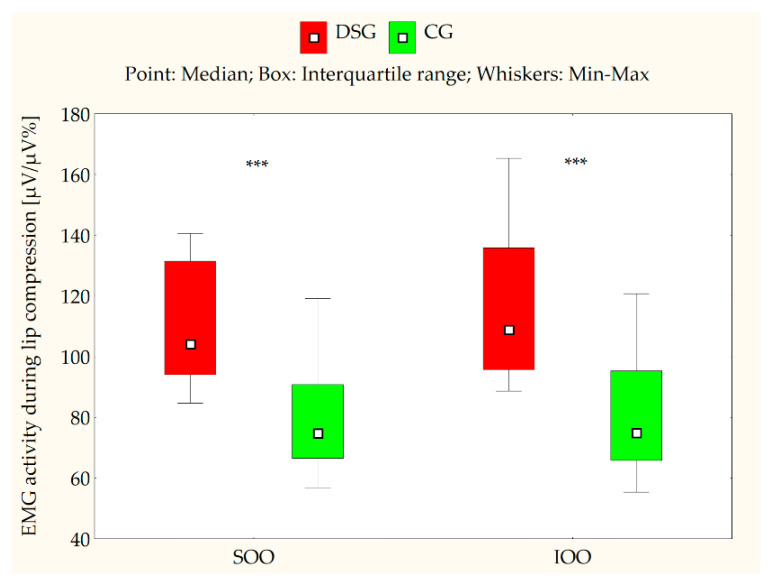
EMG activity of the superior orbicularis oris (SOO) and inferior orbicularis oris (IOO) muscles (µV/µV%) during lip compression in the Down syndrome group (DSG) and the control group (CG); *** *p* < 0.001.

**Figure 4:**
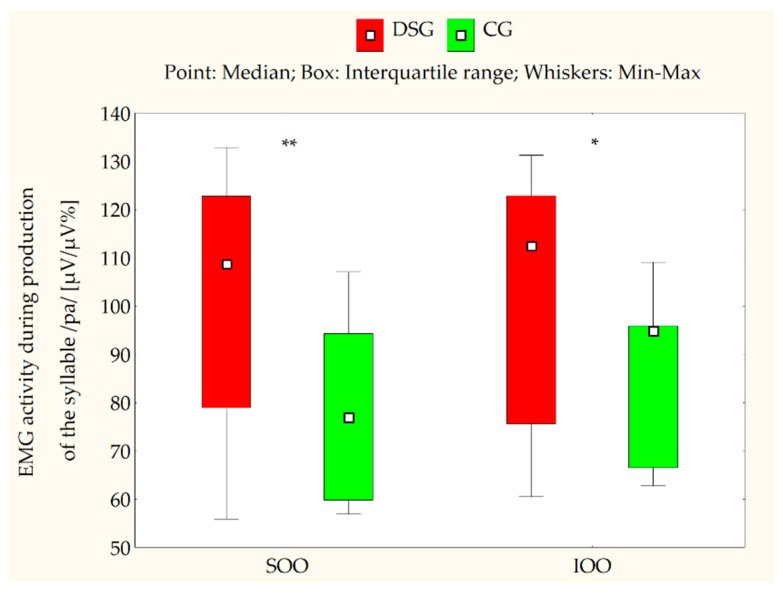
EMG activity of the superior orbicularis oris (SOO) and inferior orbicularis oris (IOO) muscles (µV/µV%) during the production of the syllable/pa/ in the Down syndrome group (DSG) and the control group (CG); ** *p* < 0.01 for SOO; * *p* < 0.05 for IOO.

**Figure 5:**
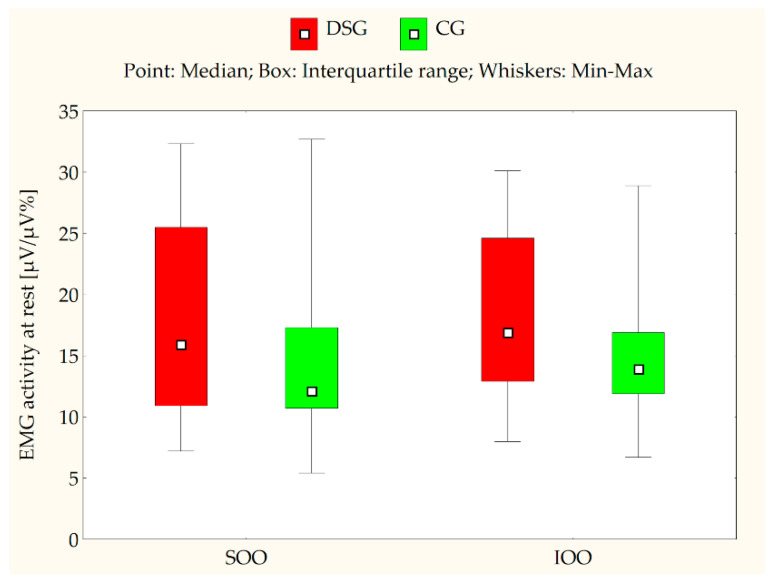
EMG activity of the superior orbicularis oris (SOO) and inferior orbicularis oris (IOO) muscles (µV/µV%) at rest in the Down syndrome group (DSG) and the control group (CG); *p* > 0.05.

Table 2 and Table 3 present the EMG measurements of the SOO and IOO muscles for both the DS and control groups at clinical rest, during lip compression and saliva swallowing, as well as when producing the syllable/pa/. An analysis of the electromyographical measurements revealed that the median values for both SOO and IOO muscle electrical activity were significantly higher in certain conditions, such as during saliva swallowing (*p* < 0.001) (Figure 2), lip compression (*p* < 0.001) (Figure 3), and the production of the syllable/pa/ (*p* = 0.008 for SOO and *p* = 0.016 for IOO) (Figure 4) in the subjects with DS compared with the healthy subjects. No statistically significant differences were observed between the DS and control groups in terms of the electrical activity of the analyzed muscles at clinical rest (*p* > 0.05) (Figure 5).

The EMG values of the SOO and IOO muscles during saliva swallowing in the DS group depending on the different clinical characteristics, such as atypical swallowing (presence of atypical swallowing vs. no diagnosis of atypical swallowing), mode of breathing (mouth breathing vs. nasal breathing), vertical overlap (anterior open bite vs. ≥0 mm vertical overlap), overjet (anterior crossbite vs. ≥0 mm overjet), a posterior crossbite (presence of posterior crossbite vs. no diagnosis of posterior crossbite), and a lateral open bite (presence of lateral open bite vs. no diagnosis of lateral open bite) are presented in Table 4 and Table 5. Statistical analysis revealed that the EMG potentials of the superior and inferior orbicularis oris muscles were significantly higher in DS subjects with atypical swallowing (*p* < 0.001), an anterior open bite (*p* = 0.028 for SOO and *p* = 0.040 for IOO), and an anterior crossbite (SOO—*p* = 0.043 and IOO—*p* = 0.036) (Table 4 and Table 5).

## 4. Discussion

The aim of this study was to analyze the electrical activity of the superior and inferior orbicularis oris muscles in special needs patients diagnosed with DS and to compare these EMG values with those of healthy subjects. The EMG analysis demonstrated significantly higher electrical signals for the SOO and IOO muscles during experimental tasks, such as saliva swallowing, lip compression, and when producing the syllable/pa/ in children with DS than in controls.

As was mentioned earlier, information regarding the EMG activity of the orbicularis oris muscle in individuals with DS is limited. For this reason, it is difficult to compare our findings with others. Nevertheless, our study results can be assessed alongside the outcomes of Nęcka et al. [9]. The authors measured the electrical activity of the superior orbicularis oris muscle in 22 subjects with DS and 23 healthy subjects with marked hypotonia of the facial muscles in various conditions, such as when the mandible is at rest, the lips are placed in a whistling position, and during maximum intercuspation. They found the electromyography signals of the SOO muscle when the lips are placed in a whistling position were significantly higher in individuals with Down syndrome compared to a neurotypical group of subjects. The authors concluded that patients with DS require additional contraction of the lip muscles to close the mouth. It was suggested that the increased sEMG potentials when the lips are in a whistling position are a result of the patient consciously tightening the muscles.

When interpreting the study results it is important to emphasize all individuals with DS had clinically assessed lip incompetence. In this context, the finding of the present research is that the higher EMG activity observed in the superior and inferior orbicularis oris muscles in children with IL during some of the tasks performed may imply a higher muscular effort due to the need to seal the lips during these functional conditions. Many previous studies showed a pattern of higher EMG activity in the orbicularis oris muscle in healthy subjects with lip incompetence than in subjects with a normal lip seal [19,20,21,22,23]. Gustafsson and Ahlgren [19] found significantly higher EMG recordings of the upper lip during such tasks as lip closure, chewing, and swallowing in children with an abnormal lip seal than in subjects with lip competence. Gamboa et al. [20] analyzed the electrical activity of the SOO and IOO muscles in 40 subjects aged between 17 and 27 both with and without lip competence at rest, as well as while speaking, swallowing, forced deep breathing, maximal voluntary clenching, and chewing. They observed that SOO and IOO muscle electrical activity was significantly higher in patients with IL than in individuals with CL during swallowing and forced deep breathing. Moreover, the authors found significantly higher EMG potentials in the SOO muscle while speaking and in the IOO muscle when chewing in subjects with lip incompetence. Their findings support the hypothesis that greater muscular effort was needed to seal the lips. Tomiyama et al. [21] showed that subjects with IL presented higher EMG activity at rest and when chewing with the lips in contact compared with those with CL. They suggested that individuals with lip incompetence have difficulty chewing due to an inability to achieve lip seal, which affects their masticatory function. What is more, Szyszka-Sommerfeld et al. [15] compared the electromyographical values of the SOO muscle during saliva swallowing in children with congenital anomalies, such as bilateral complete cleft lip and palate (BCCLP) and an abnormal lip seal with those of BCCLP children and lip incompetence. They observed significantly higher EMG potentials in children with BCCLP and an abnormal lip seal. Lipari et al. [22] evaluated the EMG activity of the SOO and IOO muscles in 40 children with CL or IL during the following tasks: At rest, speaking, swallowing, and puffing out the cheeks. They found that the EMG activity of the upper and lower lips in children with an abnormal lip seal was higher when puffing out the cheeks than in subjects with competent lips.

On the other hand, 66.7% of the DS subjects had atypical swallowing and 53.3% were predominantly mouth breathing. Moreover, children with DS had various types of malocclusion. Since our research included only subjects with Class I occlusion, the most frequent malocclusions among the DS subjects were posterior crossbites (66.7%), followed by anterior crossbites and anterior open bites (33.7%). In light of the above, it should be remembered that all these factors may result in variations in the electromyographical pattern of the SOO and IOO muscles. To discuss this issue, we also analyzed the electrical potentials of the superior and inferior orbicularis oris muscles during saliva swallowing in the DS group. An analysis of the EMG values showed that the EMG potentials of the SOO and IOO muscles were significantly higher during saliva swallowing in children with atypical swallowing. In this respect, our results are in agreement with Störmer and Pancherz [24]. Störmer and Pancherz [24] assessed the EMG potentials of the upper and lower lips in individuals with an open bite and atypical swallowing patterns and in subjects with normal swallowing in the following conditions: When swallowing saliva and water, chewing, and maximum biting in an intercuspal position. The authors found higher EMG activity in the perioral muscles when swallowing in patients with atypical swallowing compared to the controls. Similarly, López-Soto et al. [25] observed the electrical activity of the orbicularis oris muscle was greater in patients with atypical swallowing and IL than in subjects with CL and controls. They suggested that higher sEMG activity of the lips in subjects with atypical swallowing was due to the greater effort made when swallowing. Another factor that may affect muscle electrical activity is improper occlusion [26,27,28,29]. In our study, subjects with DS and malocclusions, such as anterior open bites and anterior crossbites, had significantly higher upper and lower lip electrical activity during saliva swallowing. The impact of transversal and vertical malocclusions on muscle functioning in young healthy subjects has been confirmed in previous studies [26,27,28].

To summarize, it should be pointed out that oral health care in individuals with disabilities poses a special challenge. Hence, the knowledge of orbicularis oris muscle activity in growing children with Down syndrome could be valuable for clinical practitioners dealing with the multidisciplinary treatment of DS patients. Careful analysis of upper and lower lip EMG recordings may provide a basis for improving of treatment protocols so as to normalize lip activity. Dentists dealing with the oral health care of subjects with DS should acquire the appropriate level of oral health awareness and should be aware of the special oral health needs in these patients bearing in mind that muscle hypotonia directly affects the functioning of the stomatognathic system and affects their quality of life. In our study, hypotonia of the orbicularis oris muscle in individuals with DS and lip incompetence was confirmed by surface electromyography. Our suggestion is that increased sEMG recordings of the SOO and IOO muscles in DS subjects with lip incompetence during swallowing, lip compression, and speaking may result from the conscious tightening of these muscles by the patient so as to obtain good lip closure during these functional conditions. In this context, it is important to achieve equilibrium of the orofacial muscles and correct stomatognathic functions in subjects with DS by various methods of oral motor therapy, including orofacial myofunctional therapy. As sEMG is one of the few diagnostic tools that ensure reliable, objective, and precise assessments of muscle function by detecting their electrical signals, it can be a useful aid in monitoring and evaluating the correct progress and effectiveness of these therapies [30,31]. The advantages of sEMG is that it is non-invasive, safe, and simple [13]. These factors are important when it comes to research conducted on children. However, this method has a number of shortcomings, such as its sensitivity to imbalances in impedance, which may reduce the accuracy of EMG measurements, as well as the identification of superimposed electrical potentials from numerous muscle fibers. As a consequence, the EMG analysis is restricted to assessments of general muscle activity. Nevertheless, these problems could be solved by a fixed inter-electrode distance, a standard procedure for positioning the electrodes, and adequate quantitative analysis of the data with normalization procedures [32,33,34], as was performed in our study.

The study’s limitations, such as the small number of participants involved, should be taken heed of, while at the same time also remembering that the results can only be considered a pilot study in this field. Furthermore, we compared the EMG potentials of the SOO and IOO muscles in subjects with DS and malocclusion with those of subjects with no DS and normal occlusion. Since malocclusion is one of the factors that may result in alterations in muscle electrical activity, the results of this study should be interpreted with some restrictions. In this context, we also suggest comparing the electrical activity of the orbicularis oris muscle in DS individuals vs. healthy subjects without DS, however with malocclusion. In addition, further studies covering a larger number of subjects, as well as an EMG analysis of other muscles e.g., the masticatory muscles, are necessary to support and confirm the research findings. We are currently investigating some of these topics.

## 5. Conclusions

The results of this study demonstrated the electrical activity of the superior and inferior orbicularis oris muscles was higher in children with Down syndrome and lip incompetence compared to healthy children during saliva swallowing, lip compression, and when producing the syllable/pa/, and this may suggest greater muscular effort due to the need to seal the lips during these functional conditions. Further research is required on a larger group of subjects to confirm these study results.

## Figures and Tables

**Table 1:** The results of the clinical examinations for the DS and control groups.

Variable		DS Group Mean Age 10.1 ± 1.1		Control Group Mean Age 9.8 ± 1.0	
		*n*	%	*n*	%
Gender	Females	6	40	7	46.7
	Males	9	60	8	53.3
	Total	15	100	15	100
Lip seal	Competent	0	0	15	100
	Incompetent	15	100	0	0
	Total	15	100	15	100
Atypical swallowing	No	5	33.3	15	100
	Yes	10	66.7	0	0
	Total	15	100	15	100
Mode of breathing	Nasal	7	46.7	15	100
	Mouth	8	53.3	0	0
	Total	15	100	15	100
Facial profile	Straight	7	46.7	11	73.3
	Concave	3	20	4	26.7
	Convex	5	33.3	0	0
	Total	15	100	15	100
Vertical overlap	≥0 <3 mm	6	40	15	100
	≥3 mm	4	26.7	0	0
	Reverse (anterior open bite)	5	33.3	0	0
	Total	15	100	15	100
Overjet	≥0 <3 mm	5	33.3	15	100
	≥3 mm	5	33.3	0	0
	Negative (anterior crossbite)	5	33.4	0	0
	Total	15	100	15	100
Angle Class	I	15	100	15	100
	II	0	0	0	0
	III	0	0	0	0
	Total	15	100	15	100
Posterior crossbite	No	5	33.3	15	100
	Yes	10	66.7	0	0
	Total	15	100	15	100
Lateral open bite	No	11	73.3	15	100
	Yes	4	26.7	0	0
	Total	15	100	15	100

**Table 2:** Electrical activity of the superior orbicularis oris muscle [µV/µV%] in the DS and control groups.

Activity	DS Group						Control Group					
	*n*	Min	Q1	Mdn	Q3	Max	*n*	Min	Q1	Mdn	Q3	Max
Rest	15	7.2	10.9	15.9	25.5	32.7	15	5.4	10.7	12.1	17.3	32.3
Lip compression	15	84.8	94.1	104.1	131.5	140.6	15	56.9	66.5	74.8	90.8	119.1
Saliva swallowing	15	74.3	89.1	135.7	161.2	185.0	15	43.8	53.6	82.3	99.5	108.6
Production of the syllable/pa/	15	56.9	78.9	108.7	122.8	132.8	15	55.9	59.8	76.9	94.3	107.1

Min: Minimum, Q1: First quartile, Mdn: Median, Q3: Third quartile, Max: Maximum. The Mann–Whitney U test.

**Table 3:** Electrical activity of the inferior orbicularis oris muscle [µV/µV%] in the DS and control groups.

Activity	DS Group						Control Group					
	*n*	Min	Q1	Mdn	Q3	Max	*n*	Min	Q1	Mdn	Q3	Max
Rest	15	8.0	12.9	16.9	24.6	30.1	15	6.7	11.9	13.9	16.9	28.9
Lip compression	15	88.7	95.8	108.9	135.8	165.3	15	55.4	65.8	74.9	95.4	120.7
Saliva swallowing	15	78.4	99.1	141.2	162.8	176.9	15	42.8	55.5	75.5	99.4	109.8
Production of the syllable/pa/	15	62.8	75.6	112.5	122.9	131.3	15	60.5	66.5	94.8	95.9	109.1

Min: Minimum, Q1: First quartile, Mdn: Median, Q3: Third quartile, Max: Maximum. The Mann–Whitney U test.

**Table 4:** Electrical activity of the superior orbicularis oris muscle [µV/µV%] during saliva swallowing in the DS group.

Variable		*n*	Min	Q1	Mdn	Q3	Max
Atypical swallowing	No	5	74.3	78.4	79.1	89.1	98.8
	Present	10	127.9	135.7	145.0	169.7	185.0
Mode of breathing	Nasal	7	74.3	79.1	98.8	135.7	161.2
	Mouth	8	78.4	132.2	145.0	171.3	185.0
Vertical overlap	≥0 mm	10	74.3	79.1	89.1	98.8	135.7
	Reverse (anterior open bite)	5	78.4	128.4	145.0	169.7	185.0
Overjet	≥0 mm	10	98.8	98.8	99.1	100.1	100.1
	Negative (anterior crossbite)	5	145.3	161.2	162.8	164.8	176.9
Posterior crossbite	No	5	74.3	79.1	89.1	135.7	141.2
	Present	10	78.4	127.9	142.3	169.7	185.0
Lateral open bite	No	11	74.3	79.1	128.4	141.2	172.8
	Present	4	127.9	138.3	159.2	177.4	185.0

Min: Minimum, Q1: First quartile, Mdn: Median, Q3: Third quartile, Max: Maximum. The Mann–Whitney U test.

**Table 5:** Electrical activity of the inferior orbicularis oris muscle [µV/µV%] during saliva swallowing in the DS group.

Variable		*n*	Min	Q1	Mdn	Q3	Max
Atypical swallowing	No	5	78.4	89.1	98.8	99.1	100.1
	Present	10	132.8	141.2	153.3	164.8	176.9
Mode of breathing	Nasal	7	78.4	89.1	99.1	161.2	164.8
	Mouth	8	100.1	135.4	143.3	167.8	176.9
Vertical overlap	≥0 mm	10	78.4	89.1	98.8	99.1	164.8
	Reverse (anterior open bite)	5	100.1	137.9	144.5	162.8	176.9
Overjet	≥0 mm	10	74.3	74.3	79.1	98.8	98.8
	Negative (anterior crossbite)	5	135.7	135.9	148.7	161.2	185.0
Posterior crossbite	No	5	78.4	89.1	99.1	135.7	164.8
	Present	10	98.8	132.8	144.5	162.8	176.9
Lateral open bite	No	11	78.4	98.8	132.8	161.1	172.8
	Present	4	137.9	140.8	144.5	162.8	176.9

Min: Minimum, Q1: First quartile, Mdn: Median, Q3: Third quartile, Max: Maximum. The Mann–Whitney U test.

## Data Availability

The datasets used to support the conclusions of this article are included within the article. Access to other data will be considered by the corresponding author upon request.
